# Bending the Curve: Institutional Factors Associated with Graduation Rates

**DOI:** 10.1057/s41307-023-00304-5

**Published:** 2023-02-27

**Authors:** Juliana de Castro Galvao, Frederick Tucker, Paul Attewell

**Affiliations:** 1 Department of Sociology, The Graduate School and University Center of the City University of New York

**Keywords:** degree completion, enrollment, evaluation, higher education, selectivity

## Abstract

For decades, educators and policy makers have decried low graduation rates at U.S. colleges, advocating policies and making investments to improve graduation. We analyze a decade of Integrated Postsecondary Education Data System (IPEDS) data for four-year colleges to investigate how much institutions have improved their graduation rates from 2008 through 2018, once controlling for institutional and student body characteristics. We find substantial improvement to graduation rates at public colleges, modest improvement at private not-for-profits, and a decline in graduation at the for-profit sector. We then investigate whether improvements to graduate rates are associated with variation in student-body composition, selectivity, and institutional expenditures, using pooled cross-sectional, Prais-Winsten, and college fixed effect models. We find that most between-college variation in graduation rates over time reflects variation in the composition of a college’s student body and in instructional expenditures. Our Bending the Curve metric utilizes the cross-sectional models to calculate predicted graduation rates for each college and determines how much they exceeded or failed to meet expectations. Unadjusted graduation measures, such as IPEDS’ rates that fail to adjust for these compositional factors, are poor indicators of institutional effectiveness and can mislead stakeholders who use them as an indicator of college performance.

Low degree-completion rates have been a feature of American higher education for a half-century or more. As long ago as 1971, a national report bemoaned “the surprisingly large and growing number of students who voluntarily drop out of college” (US HEW, 1974, xi). What is sometimes characterized as a current graduation crisis is by no means a recent phenomenon. However, public awareness and concern over non-completion have increased over time. In the last decade or so, philanthropies, think-tanks, and policymakers have been urging colleges to prioritize timely completion, including providing funding for innovations and programmatic changes aimed at increasing undergraduate retention and completion (Complete College America, 2020; Gates Foundation, 2020; IHEP, 2020; Lumina Foundation, 2020), as well as performance-based funding for public university systems.

In this paper, we ask whether graduation rates at 4-year colleges have indeed improved over the last decade, and whether there is variation by sector and selectivity level. We also determine what characterizes those institutions with higher graduation rates, identifying factors associated with institutional improvement over the last decade. Previous studies have either analyzed cross-sectional data (Astin, 1993, 1997; Mortenson, 1997a, 1997b; Scott, Bailey & Kienzl, 2006) to determine between-college effects on graduation, or panel data to analyze within-college effects (Zhang, 2009). We present alternative statistical models that predict colleges’ graduation rates over time: using fixed-effects models for within-college comparisons, panel models that estimate between-colleges effects, and Prais-Winsten regression models that avoid certain weaknesses of fixed-effect models (Huo, Nelson & Stevens, 2008). Our Bending the Curve metric uses cross-sectional OLS regression models to calculate predicted graduation rates for individual colleges, determining whether colleges are meeting or exceeding expectations when controlling for compositional and institutional factors.

We begin with descriptive analyses of changing graduation rates at 4-year colleges nationwide from 2008 to 2018, finding substantial improvement to graduation rates at public colleges, modest improvement at private-non-profit colleges, and declining graduation rates at private-for-profits. In our literature review, we contextualize these improvements by outlining the history of government efforts to measure institutional graduation rates and to promote graduation through performance-based funding, as well as previous criticisms of those measures and their use.

We next turn to cross-sectional models that highlight multiple factors associated with institutional graduation rates, including the composition of a college’s student body, as well as its selectivity and educational expenditures. Together these factors account for over three-quarters of the variation in graduation rates between institutions. We demonstrate that current rankings based on colleges’ graduation rates would look quite different after adjusting for student characteristics and institutional expenditures, and highlight institutions that are doing unexpectedly well considering their circumstances.

Our fixed-effects or within-college models show an improvement in graduation rates of 2.82 percentage points on average, from 2008 to 2018. Those models also reveal that those apparent improvements in colleges’ graduation rate partly reflect shifts in student enrollment patterns and demography. The final section of the paper discusses the implications of our findings for political actors who advocate using colleges’ graduation rates for accountability and funding.

## Political Context

The Student Right-to-Know and Campus Security Act (1990) mandates that U.S. universities eligible for Title IV federal funds report graduation rates for full-time, degree-seeking students. This legislation led the National Center for Education Statistics (NCES) to create the Integrated Postsecondary Education Data System (IPEDS) that biannually surveys thousands of U.S. institutions of higher education about enrollment, graduation, student demographics, faculty characteristics, and attendance costs (NCES, 2020). The political impetus behind IPEDS was partly to aid students’ decisions regarding choice of a college. These data, however, have also become a resource for measuring institutional effectiveness (Archibald & Feldman, 2008; Bailey, 2006; Dougherty et al. 2016; Hess et al. 2009; Kelchen, 2018; Scott et al. 2006; Wade, 2019).

Politicians and NGOs—such as Lumina and the Gates Foundation (Umbricht, Fernandez & Ortagus, 2017)—have pushed to tie higher education funding to student success metrics, with Tennessee first adopting performance-based funding in 1979 (Banta et al. 1996). By 2014, thirty states had conditioned at least part of their postsecondary education budgets on student success metrics (Umbricht et al. 2017). Ever since the development of the national IPEDS reporting, national politicians have been calling for similar qualifications on federal funds (Clotfelter et al. 2010; Dougherty et al. 2016; Kelchen, 2018; Kurlaender, Carrell & Jackson, 2016).

Despite these policy initiatives, there is little evidence that performance-based funding boosts graduation rates. Analyses of Pennsylvania 4-year publics (Hillman, Tandberg & Gross, 2014), Washington community colleges (Hillman, Tandberg & Fryar, 2015), and public, 4-year, Historically Black Colleges and Universities (Boland, 2018) all found little evidence that performance-based funding resulted in increased graduation rates. Li’s analysis (2020) of the 4-year public college sector from 2003 to 2015 suggests that STEM-oriented performance funding increased the proportion and overall number of graduates with STEM degrees. These excess STEM degrees, however, were offset by decreased graduation in non-STEM majors (Li, 2020). Performance funding may help institutions shift priorities, but doesn’t seem to indirectly improve overall student outcomes by rewarding supposedly higher-performing institutions.

Shifts in institutional priorities, moreover, are not always equitable. Hillman and Corral (2017) found that per-student, state allocations to Minority Serving Institutions had decreased in performance-based funding states, compared to states that eschew these practices. Kelchen and Stedrak (2016) analyzed over 1,600 public institutions from 2003 through 2012, finding that institutions with performance-based funding collected less Pell Grant revenue, likely due to prioritizing high-income students. Umbricht and colleagues (2017) found that Indiana’s performance-based funding led to increased institutional selectivity and decreased minority enrollment. Hagood (2019) analyzed hundreds of institutions, determining that performance-based funding increased allocations to selective, doctoral, and research universities. Twenty states have since tried to combat these trends by gearing performance-based funding toward graduating poor and minority students, with mixed results (Ortagus, Kelchen, Rosinger & Voorhees, 2020). Gándara and Rutherford (2018) found that Hispanic and low-income enrollment increased at these public 4-year universities, but that Black enrollment decreased; while Kelchen (2019) found the opposite at community colleges.

Another inducement to promote graduation has taken the form of bonuses paid to colleges for every low-income or minority graduate (Ortagus et al. 2020). Indiana pays colleges $6,000 for every disadvantaged student they graduate, and $23,000 for students graduating in four years (Ortagus et al. 2020). Baum and Scott-Clayton (2013) propose paying Pell Grant recipients cash bonuses for timely graduation, a less roundabout method of aiding at-risk students.

One federal government effort to boost graduation rates is the College Scorecard website, launched in 2015 (Meyer & Rosinger, 2019). Originally intended as a method for rating institutional performance, College Scorecard quickly transformed into a consumer information website, listing each institution’s tuition, graduation rate, and alumni’s average post-college earnings (Meyer & Rosinger, 2019). According to Hurwitz and Smith (2018), this increase in consumer information has only served to increase the number of students who send standardized test scores to institutions with high alumni post-college earnings. Their findings (Hurwitz & Smith, 2018) that only White and Asian students have on average changed their application behaviors suggests that this increase in consumer information may be exacerbating already existing inequalities.

## Graduation Rates and Selection Bias

The National Center for Education Statistics (NCES 2020) requires all colleges and universities receiving or applying for Title IV funding to report graduation data, and makes this data available for public use. These unadjusted graduation rates make up the basis for most analyses, including the Chronicle of Higher Education (2019) ranking of colleges by graduation rate, as well as NCES reports, like their descriptive analysis of graduation rates and low-income enrollment (Horn & Carroll 2006).Due to the extreme variation in American postsecondary institutions, even these descriptive analyses (Chronicle of Higher Education 2019; Horn & Carroll 2006) exclude a number of institutional categories, such as Tribal and Puerto Rican colleges, specialized institutions such as theological seminaries and culinary institutes, and institutions not receiving or applying for Title IV funds, which do not have mandatory reporting requirements.

There are several pitfalls when comparing graduation rates nationally using a single performance metric. An American Council on Education white-paper (Cook & Pullaro, 2010) discusses the limits of several national postsecondary data sources, including IPEDS, the National Student Clearinghouse (NSC), and NCES’ longitudinal surveys. Cook and Pullaro (2010) point out that IPEDS measures covered only first-time, full-time students who initially enrolled in the Fall semester, leaving out part-time students, transfer students, and Spring enrollees, who together account for at least half of postsecondary students. Wade (2019) documents institutions manipulating their IPEDS profile by admitting high-SAT students in the Fall and lower-SAT students in the Spring, and by encouraging underperforming first-years to drop-out before the IPEDS reporting deadline. These actions call into question the viability of using crude effectiveness measures to hold institutions accountable to serving traditionally marginalized communities without such measures backfiring.

Scott, Bailey, and Kienzl (2006) combined institution-level data (*N*=674) from the ACT, IPEDS, and the American Survey of Colleges to examine whether public or private universities were more efficient in graduating similar students relative to institutional resources. They found significant positive effects on graduation rates for instructional expenditures, higher SAT scores and more female students, and significant negative effects for a greater percentage of older and minority students (Scott et al. 2006). Using Oaxaca decomposition methods, they found public colleges to be more efficient with their resources, graduating a higher percentage of students compared to private schools with similar resources and students.

While Scott, Bailey, and Kienzl used cross-sectional data for between-college comparisons, Zhang (2009) used panel data for a within-college analysis to determine whether state funding affects graduation rates at public four-year institutions. Analyzing IPEDS and College Board data from the 1991–1998 entering cohorts at public 4-year colleges, Zhang (2009) found that a ten percent increase in state funding per full-time student was associated with a 0.64 percentage-point increase in six-year graduation, a small but significant effect.

## Bending the Curve—Adjusted Graduation Rates

In “College retention rates are often misleading” Astin (1993) reported that student inputs accounted for the majority of the variation in retention rates at 129 institutions. He later undertook multivariate analyses of student-level data at 365 baccalaureate-granting institutions (Astin, 1997) to demonstrate that four variables—high school grades, standardized test scores, gender, and race—accounted for over a third $\left(R^{2}=0.351\right)$ of variation in graduation rates and concluded that these variables might be used to produce expected graduation rates for each institution that would give researchers a better sense of how much each institution had lived up to expectations.

Mortenson (1997a, 1997b) carried out such an analysis on 1,106 colleges, using 6-year graduation rates drawn from the U.S. News and World report. Three variables—students’ SAT scores, the percent of students who live on campus, and the percent of students enrolled part-time—accounted for nearly two-thirds of the variation ($R^{2}=\;0.6564$) in institutional graduation rates. Mortenson (1997a) concluded with a list of each institution’s actual graduation rate minus its expected graduation rate, which he suggested could better help students decide which college to attend.

Some states incorporate compositional and institutional variables into their calculations for performance-based funding, albeit using methods less sophisticated than the typical academic analysis. Florida’s state university system incorporates Pell-recipient rate, the average cost of an undergraduate degree, and the percent of first-years in the top decile of their high school graduating class to help determine which campuses will receive nearly a quarter billion dollars annually (Cintron 2019). As of 2014, Ohio allocates 100% of its postsecondary educational expenses using a performance-based funding formula that weights graduation for at-risk students, including on the basis of race and Pell recipiency (Elliott, Haynes & Jones 2021). Ward and Ost (2021) utilize a differences-in-differences approach to analyze this policy, finding no evidence that it increased graduation or retention.

The U.S. News and World Report’s college rankings (Morse & Brooks 2022) are based on a formula that uses graduation and retention rates, as well as compositional and institutional factors, but which does not include race/ethnicity or gender variables. These rankings reward colleges for selectivity and greater spending per student (Morse & Brooks 2022), as opposed to our Bending the Curve metric, which uses selectivity and per-student spending to predict graduation rates, highlighting colleges with high graduation rates despite their selectivity and financial resources or lack thereof. Thus, their rankings are more relevant to prospective students choosing between dissimilar schools; whereas our rankings are better indicators of performance, controlling for student composition and institutional resources.

## Research Objectives

This research aims to address several interrelated questions concerning American postsecondary graduation rates, the first being: was there any nationwide improvement from 2008 to 2018, and did this vary by sector? Our second research query pertains to whether there are malleable compositional and institutional characteristics associated with higher graduation rates between institutions, and within institutions over time. Our expectation is that variation in graduation rates, both between institutions and within institutions over time, will be attributable mainly to selection dynamics: students selecting whether or not and where to enroll in college, and colleges selecting students who are more likely to graduate. A follow-up objective is to use this research to create an actual-minus-predicted graduation rate for each college, which we call Bending the Curve, and which allows us to determine how well or poorly colleges are doing at graduating students when controlling for compositional and institutional factors. Finally, we argue that states that allocate higher education expenses based on unadjusted or improperly-adjusted graduation rates are incentivizing college administrations to select for students more likely to graduate, thus diminishing higher education access to already disadvantaged social groups.

## Methods

### Data

In this paper we analyze data from the Integrated Postsecondary Education Data System (IPEDS) reported by colleges on a broad array of institutional and student characteristics. Aggregated data—not individual student outcomes—are collected every semester from all postsecondary institutions that participate in the federal student financial aid programs: Title IV-eligible institutions.

We restrict our analysis in a number of ways to make our predictive model applicable to the typical American bachelor’s degree-granting institution. This includes dropping all institutions that were not primarily bachelor’s degree granting in IPEDS, and non-Title IV institutions. Similar to the Chronicle of Higher Education’s rankings (2019) and Horn and Carroll’s NCES report (2006), we excluded all Tribal and Puerto Rican colleges from all analyses. After a preliminary analysis, we noticed that most colleges that were outliers in their actual-minus-predicted graduation rates were theological seminaries. Horn and Carroll (2006) exclude all “Special Focus Institutions,” including theological seminaries, culinary, art, business and management, and health schools; whereas the Chronicle of Higher Education (2019) includes these institutions. We decided to include the “Special Focus Institutions” that met our other requirements, but dropped all colleges with the Carnegie Classification of “Special Focus Institutions–Theological Seminaries, Bible Colleges, and Other Faith-Related Institutions.” Since we created adjusted graduation rates, unlike the Chronicle of Higher Education (2019), excluding these institutional categories not only impacts our final rankings, but also alters our formula for predicting colleges’ graduation rates.

Both Horn and Carroll (2006) and The Chronicle of Higher Education (2019) excluded all institutions whose full-time, first-year cohorts had fewer than 50 students. Since we are focused on change over time and wanted to avoid extreme fluctuations in graduation rates due to small sample sizes, we expanded this exclusion to any institution with fewer than 200 such enrollees. All institutions with missing graduation data from 2008–2018, or zero percent graduation rates were purged from our final list, as was any institution that had closed as of Spring 2020, even if it had full data for the period in question. After starting with 2,426 institutions, our final sample included 1,394 BA-granting, Title IV-eligible colleges.

### Analytic Design

In order to determine whether institutions that outperform others in terms of unadjusted graduation rates will continue to outperform after controlling for their student-body composition and basic institutional characteristics, we estimate the following linear model:

(1) $$G_{i}=\beta_{0}+\beta_{1}\;K_{i}+\beta_{2}S_{i}+\;\mu$$

where: G is the graduation rate (taking values 0–100) within 6-years for the bachelor’s degree-seeking cohort for each college i. These IPEDS graduation rates are based on the cohort of first-time, full-time degree-seeking students who graduated from the same institutions as reported by IPEDS. Therefore, this model does not take into account part-time and non-first-time students, or students who graduated at different institutions (Cook & Pullaro, 2010).

K is a vector of institutional characteristics that includes: Natural logarithm of instructional expenses per full-time equivalent (FTE) enrollment; Size of Institution, measured as a 5-category variables with institutions with under 1,000 students as the reference; Degree of urbanization, measured as a 12-category variable with large cities as the reference category; and selectivity and institutional sector measured as a set of dummy variables with selective private as reference category following the classification proposed in Chetty et al. (2020) who separate institutions based on their public or private status and selectivity according to Barron’s index (2008). As such, our final data selectivity variable is composed of a 6-category variable with selective private institutions as the reference category. Although our IPEDS data does not include detailed information on students’ parental income, Chetty et al. (2020) show that there is a strong correlation between this classification scheme and enrollees’ parental income. For example, for the 1980–82 birth-cohort, 68 percent of those attending Ivy-Plus colleges—defined as the eight Ivy League colleges plus Chicago, Duke, MIT, and Stanford universities—had parents with incomes in the top quintile. We include this measure of institutional selectivity instead of using standardized test scores, such as the ACT or SAT, due to the fact that not all institutions require these test scores.^1^

S is a vector of student-body characteristics that includes: Percentage of students receiving Pell grants; Percentage of total enrollment that are women; Percentage of total enrolled students that are full-time; Percentage of total enrolled students that are Black; Percentage of total enrolled students that are Hispanic/Latinos; Percentage of total enrollment that are adults (25–64)

We compare results from the regression model in equation 1 for each year between 2008 and 2018 to determine whether variables determining graduation rates have changed or remained largely the same. We are largely concerned with predictive accuracy and not inference from coefficients.^2^

To investigate the relative importance of the predictors in equation 1 towards explaining between-institutional variation in graduation rate we use *dominance analysis*. A machine learning technique, this approach provides a distinct perspective on the relative importance of predictors (Azen & Budescu 2006; Budescu 1993). Effect sizes, which are a conventional approach to assessing variable importance, indicate the change in the dependent variable associated with a one-unit increase in a predictor. By contrast, dominance analysis estimates the proportion of a regression model’s explained variance that is attributable to each predictor, net of other predictors.

From equation 1 we calculate the predicted graduation rate for each college (i) at year (t). Our bending the curve (BC) indicator is obtained by subtracting a college’s actual average graduation rate from its predicted rate:

(2) $${BC}_{i\;=\;\;}G_{i}-\;{\hat{G}}_{i}$$

where $G_{i}$ is the unadjusted (actual) graduation rate for college i and ${\hat{G}}_{i\;}$ is the adjusted graduation rate based on our predictive model in equation 1. Negative values of BC indicate that colleges are graduating a lower proportion of students than predicted, considering their institutional and student body characteristics; conversely, positive values indicate colleges are graduating more students than expected, adjusting for their characteristics.

We first investigate period effects between 2008 and 2018 by pooling all of our cross-sectional datasets from 2008 to 2018 and running an OLS regression on the pooled sample, following the same specification as equation 1 but with additional controls for each year, with 2008 as our reference. The objective is to observe time trends in graduation rates net of shifts in institutional and student body characteristics. We present results from two types of pooled model, a simple OLS model and a generalized least-squares model that uses the Prais-Winsten (1954) transformed regression estimator to correct for serially correlated errors, where these are assumed to follow a first-order autoregressive process: AR(1).

To investigate how time-varying factors have affected graduation rates from 2008 to 2018, net of institutional-level unobserved heterogeneity, we also perform a college fixed-effects estimation, which removes time-invariant effects of college characteristics, such as institutional culture, average lecture quality, and competitive environment. The fixed-effects estimation includes dummy variables for each college i, allowing each college to have a different intercept:

(3) $$G_{it}=\beta_{1}\;K_{it}+\beta_{2}S_{it}+\alpha_{i}+\;\mu_{it}\;,t=2008\ldots 2018\;$$

Where, $G_{it}$ is the graduation rate for college i in year $t.\;\alpha_{i}$ is the unobserved college specific fixed-effect and $\mu_{it}$ is the standard error term similar to that of equation 1. The explanatory vectors are the same as equation 1, with the exception that all time-fixed variables are dropped from the model. Hence, the fixed-effect model does not include parameters for institutional size, degree of urbanization, selectivity, and institutional sector. Since these factors do not change over the time period analyzed, they are subsumed along with other unobservable time-invariant characteristics in the fixed effects dummies, allowing us to estimate the change in graduation rate for each institution as a result of changes in time-varying factors. We also performed separate fixed-effects regression analyses for public and private colleges, after dropping 22 for-profit colleges from that particular analysis.

## Results

### Graduation Rates by Sector and Selectivity

**Figure 1:**
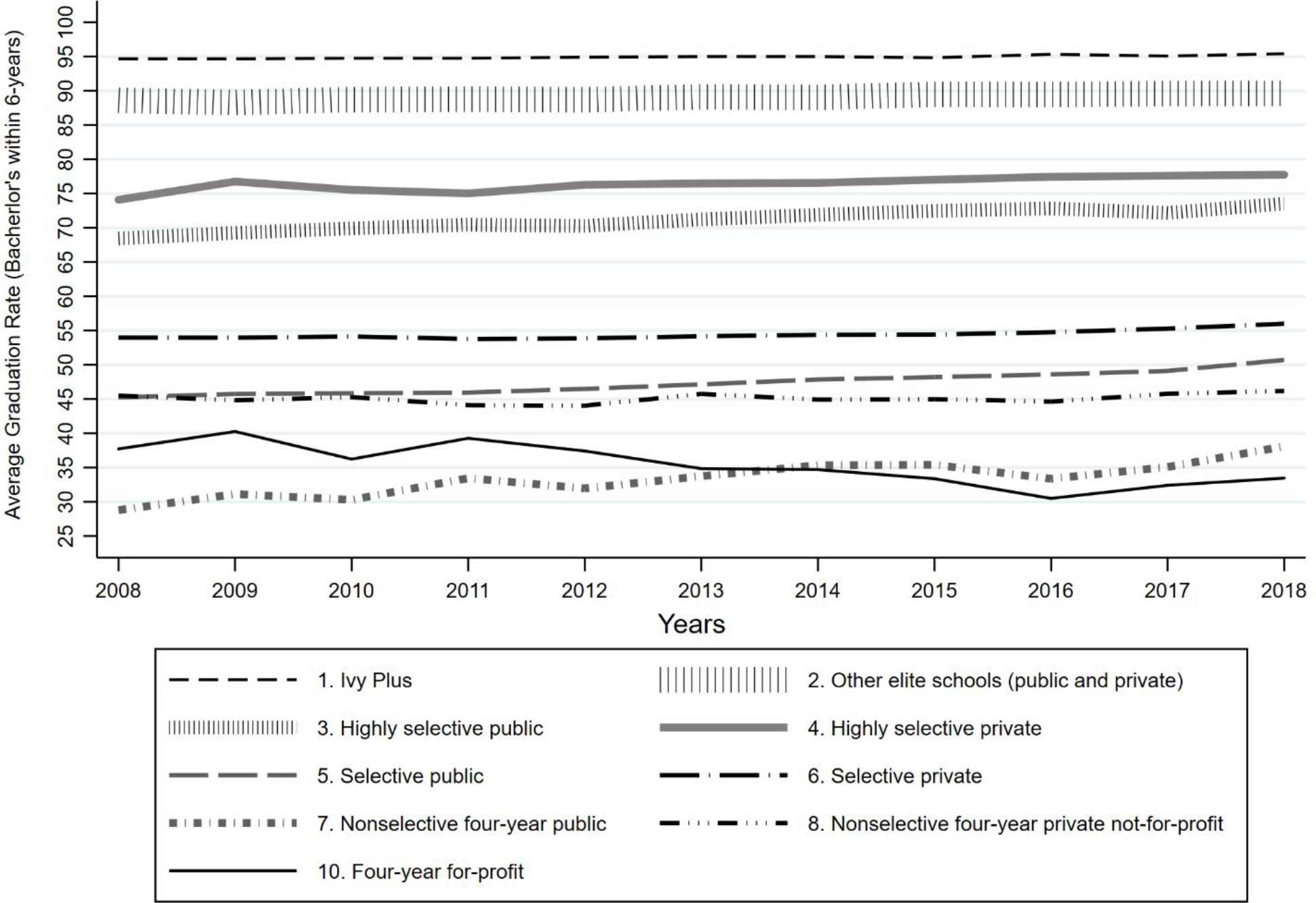
Average Unadjusted Graduation rate by Institutional Sector and Selectivity, 2008–2018

Figure 1 presents unadjusted graduation rates for each year by institutional sector and selectivity level, showing that most of the variation in graduation rates reflects differences between types of institutions rather than change over time within institutions. Between 2008 and 2018, the average 6-year graduation rate among our sample of 1,394 4-year degree-granting colleges increased modestly, averaging 54 percent in 2008 and 57 percent in 2018, with great stability year-to-year. Unsurprisingly, the highest graduating institutions are the group of twelve Ivy Plus colleges. For those institutions, the average graduation rate hovers around 95 percent, with little variation over time.

In the public sector, across different types of institutional selectivity categories, graduation rates increased by at least five percentage points. The group of Highly Selective Publics experienced an increase in graduation rates from 68.46 percent in 2008 to 73.52 percent in 2018. Selective Publics increased their graduation rates from 45.25 percent in 2008 to 50.72 percent in 2018. Nonselective Public universities had the largest increase in graduation rates for 4-year colleges, starting at 28.79 percent in 2008 and increasing to 38.09 percent by 2018.

The four-year, private, non-profit sector saw smaller increases in graduation rates. Nonselective Privates improved their graduation rates by less than one percentage point, from 45.5 percent in 2008 to 46.19 percent in 2018. Graduation rates at Selective Privates increased by two percentage points during the same time period (53.98%–56.01%). Highly Selective Privates experienced the largest increase in the private, not-for-profit sector, going from 74.1 percent in 2008 to 77.75 percent by 2018. The only 4-year institutional sector that experienced a decrease in average graduation rates over this recent decade were for-profit colleges, their graduation rate decreasing from 37.73 percent in 2008 to 33.57 by 2018.

### Student-Body Composition & Institutional Factors

In Table 1 we report results from multiple cross-sectional OLS regression models predicting the 6-year graduation rate, one model for each year from 2008 to 2018. The focus is on which institutional and student-body characteristics are associated with higher graduation rates among the 1,394 4-year degree-granting institutions, and whether these factors vary through time. The pattern is stable from year to year, and the predictive models are quite powerful (with *R*^*2*^ values around 0.75).

According to the dominance analysis for the year 2018 (Table 2), the percentage of students receiving Pell grants is the single most important factor in determining graduation rates, explaining 23.3 percent of the explained variance in graduation. The larger the percentage of the student body supported by a Pell Grant, the lower the institution’s graduation rate. In terms of effect size, in 2018, for every 10 percentage-point increase in Pell enrollment, the graduation rate decreases by about 4.1 percentage points; whereas a decade prior, in 2008, a 10 percentage-point increase in Pell enrollment was associated with a graduate rate decrease of 3 percentage points (see Table 1).

The variable that had the second largest dominance impact on graduation rates in our models is expenditure on instruction, accounting for 18.4 percent of explained graduation variance. In 2018, every percentage-point increase in instructional expenditures was associated with a 6.63 percentage point increase in graduation rate.

The third most important predictor in explaining graduation rates is college tier. College tier measures a college’s academic selectivity and is strongly correlated with undergraduates’ parental income (Chetty et al. 2020). It should therefore be viewed as an indicator of both academic and social selectivity. Table 1 reported coefficients comparing Ivy Plus and Other Elite Schools (public and private) to the reference category, Selective Private Institutions. The graduation rate advantage between the Ivy Plus and Other Elite colleges, and the Selective Privates decreased from about 16 percentage points in 2008 to 8 percentage points in 2018. By contrast, the Selective Publics graduate on average a lower proportion of their students than the Selective Privates in all years.

The fourth most important predictor (Table 2) is the proportion of adult students (25–64 years old). Table 1 shows that an increase in the percent of adult first-time students is negatively associated with graduation rates. This effect, however, is not large; in 2018, for example, even a substantial increase of 10 percentage-points in adult enrollment would be associated with only roughly 2 percentage points decrease in graduation rates. The percent of total enrollment of African-American students is also associated with lower graduation rates. This effect, however, is not statistically significant in all years, and is small in magnitude. In 2018, a college with 10 percentage-points more African-American students would have a 0.7 percentage-point lower graduation rate. A larger effect is seen for the influence of percent full-time students; for every 10 percentage-point increase in full-time students, the graduation rate is 1.4 percentage-points higher.

The smallest colleges (those with 1,000 or fewer students) tend to have the lowest graduation rates. Mid-size colleges have graduation rates of roughly 8 percentage-points higher than those of small colleges. The largest colleges (20,000 students and above) have between 11 and 14 percentage-point higher graduation rates on average than colleges with fewer than 1,000 students, holding other factors constant. With the exception of these factors and institutional expenses, other institutional characteristics do not seem to have a large impact in predicting institutional graduation rates.

Finally, colleges that enroll a larger proportion of women have slightly higher graduation rates on average, a significant but small effect, net of other factors. Colleges with a higher percentage of Hispanic students were significantly associated with a lower graduation rate in 2008; the effect sizes, however, are small and non-significant after this period until 2017. In 2017 and 2018, the effect is reversed; by those years, greater Hispanic enrollment had become associated with *higher* graduation rates.

These cross-sectional analyses showing the importance of student composition and institutional characteristics reinforce Astin (1997) and others’ argument about the importance of controlling for student-body composition when judging which institutions are more or less effective in graduating students. To highlight this for the recent period, in Table 3 we report the difference in rankings between colleges that we obtain using only actual (unadjusted) graduation rates for the last eleven years, as opposed to using a predictive model to adjust rates.

### Who is Bending the Graduation Curve?

Panel A of Table 3 lists the top ten colleges by actual graduation rate. They are mostly elite, highly selective, private universities. If we look at the right-most column in Panel A, however, we see how these colleges rank within the 1,394 colleges, using the expected graduation rate, given their student and institutional characteristics. None of these elite institutions would be in the top-200 in terms of actual-minus-predicted graduation rates. Harvard University for example, would drop from 2^nd^ place to 223^rd^ if we were to use the adjusted rank based on our predictive model. Yale University, with the second largest endowment, would drop from 3^rd^ to 621^st^.

In Panel B of Table 3 we report the top ten colleges in terms of graduating more students than predicted. These universities have relatively low actual graduation rates but are nevertheless very efficient at graduating their students considering their institutional and student-body characteristics. For example, Robert Morris University in Illinois graduates 76 percent of their students; considering their institutional and student body characteristics, however, we would expect them to graduate only 38 percent of their students. Colleges like this with large positive disparities between expected and actual graduation rates should be studied to identify best practices and other factors associated with their superior performance.

The large disparity between observed colleges’ graduation rate and the expected rate adjusted for student composition and institutional characteristics also implies that websites such as “The College Report Card” that simply report unadjusted rates can mislead policy makers who use them to reward financially more productive or punish less effective colleges.

### What Factors are Associated with Bending the Graduation Curve?

Table 4 presents the results from several regression models. Here we focus on the results from the model with the Prais-Winsten specification which corrects for serially correlated errors. These models do not include college fixed-effects but do include controls for year. This allows us to observe whether graduation rates have been increasing over time, net of controls. Looking at the dummy-year variables we can see in 2018 that college graduation rates were on average only 2.82 percentage points higher than in 2008.

In Table 5, we examine within-college variation in average graduation rates from 2008 to 2018 in a fixed-effects model. The left-hand column reports for public institutions, and the right hand-column for private, not-for-profit institutions. The rho parameter shows that the great majority of variation in college graduation rates observed from 2008 and 2018 is attributable to differences between institutions rather than within institutions. For public 4-year colleges, 95.2 percent of the variance in graduation rates is due to differences across panels; for private, not-for-profits it is 89.1 percent. The fixed-effects specification can nevertheless shed light on factors that explain change within institutions observed over the period.

While the percent of students receiving Pell Grants was a strong predictor of graduation rates in our cross-sectional models, changes in the proportion of students receiving Pell over time within colleges have no statistically significant impact once college fixed-effects are accounted for. Instructional expenditures, however, remain a statistically significant predictor of graduation rates within public institutions, as it was in cross-sectional models. On average, an increase of only 1 percent in instructional expenditures is associated with a 2.3 percentage-point increase in graduation rates for public colleges. By contrast, for private, not-for-profit institutions we find no statistically significant impact of instructional expenditures per student on graduation rates.

The percent of women enrolled is only statistically significant for private institutions. The effect size of the coefficient, moreover, is small; even a 10 percentage-point increase in female students over time would increase graduation rates by only 1.1 percentage points. Increases in Hispanic enrollment are statistically significant for both public and private institutions and have a positive impact on graduation rates across time, within institutions. For public colleges, a 1 percentage-point increase in Hispanic enrollment on average increases graduation rates within colleges by about 0.46 percentage-points over the decade; for private institutions, the increase is about 0.20 percentage points.

## Discussion & Conclusion

### Misleading Graduation Rates

We draw two main conclusions from our analyses. First, there have been modest increases in graduation rates at baccalaureate granting colleges from 2008 through 2018, concentrated especially at public institutions (Figure 1). In our fixed-effects longitudinal models, the most important factors in explaining this improvement in publics were increased instructional expenditures, an increase in the proportion of Hispanic students, and decreased enrollment in adults 25-plus years-old. Second, judging a college’s institutional effectiveness based on its unadjusted graduation rate—as is the case on the federal government’s College Scorecard website and some states’ performance-based funding models—will mislead politicians and higher-education administrators as to institutional effectiveness. In performance-based funding states, the imperative to raise graduation rates without accounting for the right compositional and institutional factors may reward colleges that select for the most advantaged students and punish already under-resourced institutions that serve primarily disadvantaged students.

The IPEDS and College Scorecard systems place a considerable reporting burden and substantial costs on colleges and a major expenditure for the federal government itself. One rationale is that graduation data over time allow colleges and policymakers to see whether efforts at improving graduation are having an impact. A second rationale is that these data provide policymakers information about colleges’ efficacy or performance that can be used for accountability.

Our analyses echo and update those of earlier scholars in showing that a college’s IPEDS graduation rate largely reflects the mix of students who attend that college, plus its level of instructional expenditures. Politicians and higher education administrators who compare unadjusted graduation rates across several colleges could easily be misled into believing that this is a measure of colleges’ quality of education (Kim & Shim, 2019), or its relative efficacy in graduating students, when in fact graduation rate variation largely reflects between- and within-college variation in the composition of their student bodies, due to selection biases stemming from college selectivity and student enrollment patterns. Policies that view a college’s graduation rate as a relative measure of college performance or efficacy, for accountability purposes, make a similar attribution error (e.g., Hagood, 2019; Hess et al. 2009; Kelchen & Stedrak, 2016; Umbricht et al. 2017). Using variation in an individual college’s graduation rate over time as an indicator of improved effectiveness in graduating its students has similar pitfalls; institutions can rapidly change their recruiting tactics and selectivity (Dougherty et al. 2016; Kelchen, 2018; Umbricht et al. 2017). Chetty et al. (2020) reported that some colleges with improved graduation rates may have achieved this by reducing their enrollment of minority or lower-income students over time. We did not find evidence for that process in our college fixed-effects models. Increases in Hispanic enrollment, along with decreases in older students were associated with improved graduation rates.

### Adjusted Graduation Rates

In our analysis of IPEDS graduation rates from 2008 through 2018, we found substantial improvement for public colleges at every level of selectivity. The most improvement came from non-selective publics, which had the worst graduation rates to begin with, indicating that the efforts of politicians and philanthropic organizations have caused these institutions to prioritize improving graduation rates. Like Scott, Bailey, and Kienzl (2006) and their analysis of public, four-year colleges, we found that instructional expenditures have a significant, positive effect on graduation rates, ranking second in importance in our dominance analysis. Zhang (2009) also found that state expenditures for public universities were positively and significantly associated with graduation rates. Backed by ample evidence across multiple decades, increasing state expenditures for public universities is perhaps the least complex mechanism—administratively, if not politically—for increasing graduation rates without directly altering the student body composition.

Another complex policy puzzle regarding graduation rates concerns Pell Grants. Our dominance analysis (Table 2) finds that the percentage of a student body receiving Pell Grants is the most important factor affecting institutional graduation rates in 2018, just not in the desired direction. Our OLS models (Table 1) show that every additional percentage-point increase in the student body receiving Pell Grants is associated with a 0.3 to 0.45 percentage-point reduction in institutional graduation rates. Performance-based funding that utilizes unadjusted graduation rates disincentivizes institutions from taking Pell Grant students (Kelchen & Stedrak, 2016). States planning on adopting performance-based funding should take Ohio’s lead (Elliott, Haynes & Jones 2021) and make sure to adjust their graduation rates for Pell recipiency rates.

Our adjusted graduation rate—Bending the Curve metric—by controlling for compositional and institutional factors, gauges how well schools perform given their students and resources. Whereas the U.S. News and World Report rankings reward colleges for selecting academically stronger students and having more financial resources, our Bending the Curve metric penalizes more selective, well-funded colleges if these factors do not result in higher graduation rates. We do not wish to suggest that students use our Bending the Curve metric instead of U.S. News and World Report rankings when trying to decide which college to attend among many dissimilar choices. Our Bending the Curve rankings are meant to highlight colleges that do unexpectedly well given their circumstances, and call into question whether it is wise to create a system of financial rewards that incentivizes selection bias.

## Figures and Tables

**Table 1:** Ordinary Least Squares Cross-sectional Models Predicting Graduation Rate within 6-years for Bachelor’s Degree Seeking Cohort, 2008–2018

	2008	2009	2010	2011	2012	2013	2014	2015	2016	2017	2018

Percent Receiving Pell Grant	−0.301***	−0.394***	−0.352***	−0.378***	−0.400***	−0.413***	−0.444***	−0.432***	−0.425***	−0.447***	−0.417***
	(0.038)	(0.041)	(0.037)	(0.036)	(0.030)	(0.031)	(0.028)	(0.031)	(0.031)	(0.035)	(0.030)
Ln Instruction Expenses per FTE	5.782**	6.819***	6.461***	7.296***	6.848***	6.895***	6.806***	7.382***	7.467***	6.563***	6.633***
	(1.866)	(1.037)	(1.080)	(0.981)	(0.885)	(0.846)	(0.774)	(0.816)	(0.790)	(0.811)	(0.771)
Percent of total enrollment Black	−0.095***	−0.055*	−0.100***	−0.093***	−0.070***	−0.040	−0.038	−0.050*	−0.052*	−0.042	−0.073***
	(0.026)	(0.024)	(0.023)	(0.024)	(0.020)	(0.021)	(0.021)	(0.022)	(0.022)	(0.024)	(0.022)
Percent Enrollment Hispanic	−0.089*	−0.037	−0.025	−0.025	0.013	0.004	0.0574	0.016	0.0529	0.0875**	0.0583*
	(0.040)	(0.038)	(0.035)	(0.040)	(0.034)	(0.030)	(0.031)	(0.029)	(0.029)	(0.029)	(0.027)
Percent Full-time Enrollment	0.105**	0.125***	0.143***	0.131***	0.123***	0.127***	0.145***	0.151***	0.129***	0.145***	0.136***
	(0.034)	(0.028)	(0.037)	(0.033)	(0.029)	(0.026)	(0.024)	(0.026)	(0.024)	(0.024)	(0.025)
Percent Enrollment Women	0.131***	0.081**	0.089**	0.120***	0.114***	0.140***	0.155***	0.144***	0.129***	0.118***	0.083***
	(0.032)	(0.031)	(0.027)	(0.026)	(0.024)	(0.026)	(0.024)	(0.024)	(0.025)	(0.025)	(0.024)
Adult Enrollment (25 – 64)	−0.254***	−0.179***	−0.202***	−0.183***	−0.195***	−0.180***	−0.177***	−0.166***	−0.192***	−0.188***	−0.199***
	(0.037)	(0.034)	(0.039)	(0.035)	(0.033)	(0.031)	(0.027)	(0.028)	(0.029)	(0.029)	(0.033)
*Tier (Reference: Selective Private)*											
Ivy Plus	15.590***	12.570***	10.830***	8.698***	9.738***	9.762***	9.437***	8.225***	7.923***	8.848***	8.474***
	(3.222)	(2.107)	(2.069)	(1.930)	(1.944)	(1.843)	(1.943)	(2.041)	(1.995)	(2.018)	(1.863)
Other Elite Schools (Public and Private)	15.530***	12.03***	10.790***	9.380***	9.976***	10.210***	9.519***	9.047***	8.843***	9.155***	8.403***
	(2.372)	(1.278)	(1.237)	(1.150)	(1.137)	(1.120)	(1.068)	(1.123)	(1.120)	(1.099)	(1.035)
Highly Selective Public	2.602	1.540	−0.431	−0.072	1.047	1.395	1.763	1.391	1.152	0.0824	0.901
	(1.572)	(1.510)	(1.377)	(1.266)	(1.229)	(1.176)	(1.227)	(1.180)	(1.174)	(1.471)	(1.371)
Highly Selective Private	6.235***	7.510***	5.367***	5.171***	6.450***	6.031***	5.804***	5.647***	5.629***	5.282***	4.795***
	(1.604)	(1.319)	(1.143)	(1.199)	(1.093)	(1.069)	(1.024)	(1.089)	(0.995)	(1.077)	(0.957)
Selective Public	−9.592***	−9.191***	−10.83***	−10.27***	−8.659***	−8.936***	−7.716***	−7.424***	−7.796	−7.787***	−6.665***
	(0.903)	(0.898)	(0.805)	(0.753)	(0.733)	(0.750)	(0.714)	(0.748)	(0.750)	(0.793)	(0.759)
Nonselective four-year Public	−14.920***	−14.14***	−15.60***	−12.23***	−12.33***	−11.90***	−10.67***	−9.189***	−12.25***	−11.97***	−9.351***
	(2.468)	(1.911)	(1.948)	(1.712)	(2.050)	(2.248)	(1.841)	(1.903)	(1.851)	(1.849)	(1.656)
Nonselective four-year private not-for-profit	−1.521	−1.852	−0.859	−1.658	−2.151	−1.071	−2.067	−2.080	−2.968*	−3.371*	−3.817**
	(2.055)	(1.890)	(1.818)	(1.687)	(1.481)	(1.443)	(1.297)	(1.454)	(1.313)	(1.399)	(1.317)
Four-year for-profit	−4.465	2.195	−1.228	0.704	0.428	−4.394	−1.750	−3.006	−4.870*	−2.081	−2.870
	(2.917)	(2.459)	(2.939)	(2.415)	(2.070)	(2.752)	(2.200)	(2.581)	(2.369)	(2.776)	(2.418)
*Institution Size (Reference: Under 1,000)*											
1000 – 4,999	4.525***	6.437***	6.546***	6.420***	5.206***	5.393***	5.353***	4.668***	5.195***	4.149***	4.031***
	(1.339)	(1.319)	(1.358)	(1.308)	(1.061)	(1.123)	(0.996)	(1.115)	(0.989)	(1.084)	(0.987)
5,000 – 9,999	7.725***	9.372***	9.798***	9.600***	7.984***	8.126***	7.819***	7.306***	8.503***	7.627***	7.327***
	(1.566)	(1.553)	(1.584)	(1.520)	(1.284)	(1.283)	(1.175)	(1.288)	(1.201)	(1.255)	(1.178)
10,000 – 19,999	7.824***	9.570***	10.130***	10.450***	8.843***	9.739***	8.920***	7.704***	7.809***	6.798***	6.485***
	(1.698)	(1.678)	(1.754)	(1.641)	(1.406)	(1.383)	(1.277)	(1.424)	(1.289)	(1.380)	(1.283)
20,000 and above	11.000***	13.140***	14.540***	14.430***	12.880***	14.00***	13.27***	13.13***	13.76***	12.73***	12.24***
	(1.877)	(1.781)	(1.808)	(1.715)	(1.492)	(1.451)	(1.354)	(1.503)	(1.400)	(1.499)	(1.391)
*Urbanicity (Reference: City (Large))*											
City (Midsize)	0.026	−0.802	0.512	0.781	1.107	0.489	0.689	0.009	−0.296	−0.412	−0.549
	(1.152)	(1.087)	(1.027)	(1.047)	(0.891)	(0.873)	(0.870)	(0.920)	(0.841)	(0.881)	(0.847)
City (Small)	−0.312	0.105	0.226	0.702	0.594	0.873	0.0562	−0.579	−0.343	0.249	−0.425
	(1.144)	(1.085)	(0.986)	(0.950)	(0.876)	(0.857)	(0.838)	(0.859)	(0.861)	(0.887)	(0.804)
Suburb (Large)	2.592*	1.821	2.555**	2.685**	1.958*	1.889*	1.464*	1.420	1.686*	1.852*	1.680*
	(1.031)	(0.941)	(0.974)	(0.860)	(0.784)	(0.783)	(0.734)	(0.779)	(0.791)	(0.790)	(0.743)
Suburb (Midsize)	−0.620	−1.393	−0.123	0.744	0.906	1.251	0.494	0.868	−0.684	0.571	−0.485
	(1.763)	(1.676)	(1.526)	(1.402)	(1.295)	(1.452)	(1.236)	(1.316)	(1.445)	(1.494)	(1.576)
Suburb (Small)	4.608*	4.172*	5.668***	3.477*	3.124*	2.818	2.389	0.534	1.099	1.300	0.395
	(1.962)	(1.687)	(1.700)	(1.733)	(1.574)	(1.617)	(1.443)	(1.600)	(1.635)	(1.533)	(1.728)
Town (Fringe)	−0.116	1.123	2.297	4.693**	1.391	1.584	0.992	0.677	1.003	−0.818	−0.836
	(1.796)	(1.925)	(1.922)	(1.788)	(1.430)	(1.268)	(1.317)	(1.317)	(1.345)	(1.388)	(1.284)
Town (Distant)	3.220*	0.448	1.350	1.717	2.086*	3.803***	2.279*	1.503	1.440	1.409	0.0914
	(1.546)	(1.077)	(1.072)	(0.986)	(1.031)	(1.026)	(0.930)	(0.972)	(0.951)	(0.998)	(0.916)
Town (Remote)	−0.971	−2.003	−1.515	−1.140	−1.403	−0.474	−2.210*	−3.624**	−3.314**	−3.354**	−4.143***
	(1.292)	(1.182)	(1.186)	(1.107)	(1.084)	(1.035)	(1.071)	(1.131)	(1.132)	(1.106)	(1.098)
Rural (Fringe)	−0.493	−1.049	−0.064	2.276	−0.232	1.203	−0.130	−1.348	−2.811	−0.177	−1.879
	(1.480)	(1.322)	(1.524)	(1.278)	(1.796)	(2.076)	(1.798)	(2.013)	(1.875)	(2.209)	(1.873)
Rural (Distant)	−1.833	2.029	−0.005	−1.001	−1.841	−0.885	−4.317*	−3.569	−1.248	−2.511	−2.952
	(2.273)	(2.088)	(2.282)	(1.971)	(2.121)	(2.284)	(2.025)	(2.248)	(1.742)	(2.027)	(2.033)
Rural Remote	2.149	3.337	1.846	1.529	−0.804	1.546	4.842*	−0.504	−2.106	7.465	4.656
	(2.637)	(2.447)	(2.892)	(2.611)	(2.226)	(2.430)	(2.216)	(2.778)	(2.878)	(5.192)	(5.128)

Constant	−2.439	−10.880	−8.268	−15.34	−9.026	−12.16	−12.53	−16.73*	−15.64*	−6.872	−3.208
	(17.03)	(9.985)	(10.46)	(9.996)	(8.634)	(8.184)	(7.487)	(7.901)	(7.926)	(7.842)	(7.523)
*N*	1394	1394	1394	1394	1394	1394	1394	1394	1394	1394	1394
R^2^	0.676	0.706	0.734	0.762	0.783	0.777	0.790	0.779	0.792	0.773	0.785

Standard errors in parentheses

* p<0.05

** p<0.01

*** p<0.001

**Table 2:** Analysis of Dominance 2018 Graduation Rate, Ordinary Least Squares Regression Model

	Dominance Stat.	Standardized Domin Stat.	Rank
% Receiving Pell Grants	0.222	0.283	1
Ln of Instructional Expenses	0.144	0.184	2
Tier	0.138	0.176	3
% Undergrad enroll 25–64	0.093	0.118	4
% Black	0.071	0.091	5
% Full-time enrollment	0.052	0.066	6
Inst. Size	0.033	0.042	7
Locale (degree of Urbanization)	0.025	0.032	8
% Women	0.004	0.005	9
% Hispanic	0.004	0.005	10

Overall Fit Statistic	0.785		
*N*	1,394		

**Table 3:** 2018 Bachelor’s degree within 6-years Actual Graduation Rate versus Rank Based on Predicted Graduation Rate

Panel A: Top 10 Institutions with Highest Graduation Rates					

Actual Rank	Actual Minus Predicted Rank	Institution	Actual Graduation Rate	Predicted Graduation Rate	US News & World Report Ranking

1	233	Harvard University	98.0	90.4	2 (Overall)
2	621	Yale University	97.0	95.7	3 (Overall)
3	594	University of Notre Dame	97.0	95.3	15 (Overall)
4	647	Princeton University	96.0	95.0	1 (Overall)
5	930	Columbia University (NYC)	96.0	99.1	3 (Overall)
6	738	Duke University	96.0	96.3	10 (Overall)
7	479	Washington and Lee University	95.0	91.8	10 (Lib Arts)
8	1070	Washington University in St Louis	95.0	100.6	19 (Overall)
9	257	Williams College	95.0	88.0	1 (Lib Arts)
10	273	Bowdoin College	95	88.30	6 (Lib Arts)
Panel B: Top 10 Institutions at Graduating more than Expected					

Actual Rank	Actual Minus Predicted Rank	Institution	Actual Graduation Rate	Predicted Graduation Rate	US News & World Report Ranking

213	1	Robert Morris University Illinois	76.0	38.2	33 (Midwest)
615	2	Everglades University	59.0	27.2	96–122 (South)
419	3	Albertus Magnus College	66.0	34.6	66 (North)
353	4	CUNY Bernard M Baruch College	69.0	43.9	16 (North)
250	5	Maine Maritime Academy	74.0	49.9	5 (North)
616	6	SAE Expression College	59.0	35.0	Unranked
271	7	Martin Luther College	73.0	50.9	Unranked
1153	8	Metropolitan State University	39.0	17.2	293–381 (Overall)
315	9	Mount Mercy University	71.0	49.5	40 (Midwest)
842	10	Clarks Summit University	51.00	30.27	Unranked

**Table 4:** Pooled OLS Regression Models

	Pooled OLS	Prais-Winsten

Percent Receiving Pell Grant	−0.394***	−0.130***
	(0.00820)	(0.00918)
Ln Instruction Expenses per FTE	6.860***	3.782***
	(0.233)	(0.337)
*Tier (Reference: Selective Private)*		
Ivy Plus	9.960***	19.75***
	(0.917)	(1.980)
Other Elite Schools (Public and Private)	10.28***	18.60***
	(0.445)	(0.935)
Highly Selective Public	1.025*	4.006***
	(0.473)	(1.050)
Highly Selective Private	5.840***	10.51***
	(0.357)	(0.789)
Selective Public	−8.657***	−8.746***
	(0.234)	(0.493)
Nonselective 4-year Public	−12.29***	−14.26***
	(0.547)	(1.233)
Nonselective 4-year private not-for-profit	−2.157***	−4.578***
	(0.336)	(0.747)
4-year for-profit	−1.936**	−7.737***
	(0.635)	(1.422)
Adult Enrollment (25 – 64)	−0.192***	−0.240***
	(0.00742)	(0.0130)
Percent of total enrollment Black	−0.0688***	−0.196***
	(0.00582)	(0.0101)
Percent Full-time Enrollment	0.136***	0.109***
	(0.00704)	(0.0118)
*Institution Size (Reference: Under 1,000)*		
1000 – 4,999	5.290***	2.695***
	(0.275)	(0.380)
5,000 – 9,999	8.339***	5.412***
	(0.356)	(0.524)
10,000 – 19,999	8.621***	6.843***
	(0.392)	(0.632)
20,000 and above	13.31***	10.97***
	(0.431)	(0.747)
*Urbanicity (Reference: City (Large))*		
City (Midsize)	0.160	−0.315
	(0.276)	(0.519)
City (Small)	0.0991	−0.557
	(0.267)	(0.526)
Suburb (Large)	1.985***	1.423**
	(0.251)	(0.501)
Suburb (Midsize)	0.268	0.164
	(0.487)	(0.860)
Suburb (Small)	2.631***	1.654
	(0.527)	(0.935)
Town (Fringe)	0.723	−0.799
	(0.446)	(0.694)
Town (Distant)	1.657***	−0.948
	(0.300)	(0.559)
Town (Remote)	−2.155***	−3.029***
	(0.347)	(0.637)
Rural (Fringe)	−0.358	−0.402
	(0.429)	(0.585)
Rural (Distant)	−1.577**	−3.774***
	(0.582)	(0.994)
Rural Remote	2.117*	−3.014
	(0.839)	(1.561)
Percent Enrollment Women	0.119***	0.0869***
	(0.00689)	(0.0143)
Percent Enrollment Hispanic	0.0152	−0.0795***
	(0.00885)	(0.0175)
*Year (Reference: 2008)*		
2009	0.102	0.310
	(0.349)	(0.173)
2010	2.059***	0.896***
	(0.353)	(0.238)
2011	3.351***	1.260***
	(0.359)	(0.283)
2012	3.111***	1.221***
	(0.359)	(0.307)
2013	2.879***	1.341***
	(0.359)	(0.324)
2014	2.828***	1.454***
	(0.360)	(0.340)
2015	2.824***	1.538***
	(0.362)	(0.353)
2016	2.587***	1.475***
	(0.362)	(0.362)
2017	2.722***	1.802***
	(0.362)	(0.370)
2018	4.107***	2.818***
	(0.365)	(0.380)
_cons	−13.65***	15.38***
	(2.239)	(3.357)

*N*	15,334	15,334
R-sq	0.755	0.609
Rho		0.775

Standard errors in parentheses

* p<0.05

** p<0.01

*** p<0.001

**Table 5:** Fixed Effects estimates of determinants of bachelor’s degree within 6-years between 2008–2018 by Sector

	Public	Private not-for-profit

Percent Receiving Pell Grant	0.00179	−0.0179
	(0.0161)	(0.0168)
Ln Instruction Expenses per FTE	2.303**	0.536
	(0.701)	(0.589)
Percent Enrollment Women	0.116	0.111*
	(0.0723)	(0.0518)
Percent Full-Time Enrollment	0.0446	0.000165
	(0.0316)	(0.0209)
Percent Enrollment Black	−0.0107	−0.0873
	(0.0438)	(0.0461)
Percent Enrollment Hispanic	0.456***	0.200***
	(0.0503)	(0.0423)
Adult Enrollment (25 – 64)	−0.183***	−0.0313
	(0.0447)	(0.0269)
Constant	17.83*	47.70***
	(8.453)	(6.399)
rho	0.952	0.891
R-Square overall	0.076	0.184
within	0.169	0.012
between	0.071	0.204
Number of Obs.	5434	9667
Number of Groups	494	881

Standard errors in parentheses

* p<0.05

** p<0.01

*** p<0.001

## Data Availability

All data analyzed in this paper is derived from Integrated Postsecondary Education Data System (IPEDS). This data is for public use, and is available at their website (NCES 2020).

## Footnotes

1. Roughly 13% of the institutions in our sample do not require test scores. Additionally, there is a high correlation between requiring test scores and the performance in these tests with our institutional selectivity variable. As such, to not lose information particularly on lower ranked institutions and following recent research we chose to include only institutional selectivity in the final models.

2. We also performed tests using 6-year lagged predictors of graduation rates. For example, 2018 graduation rates were predicted using 2013 predictors. Our models remained very similar and our meaningful conclusions did not change. As such, we opted to use contemporaneous predictors that would allow us to increase our long-term analysis.
